# MicroRNA-384 inhibits nasopharyngeal carcinoma growth and metastasis via binding to Smad5 and suppressing the Wnt/β-catenin axis

**DOI:** 10.1007/s10616-021-00458-3

**Published:** 2021-02-26

**Authors:** Xinyu Zeng, Huiqun Liao, Fusen Wang

**Affiliations:** 1 Department of Otorhinolaryngology, People’s Hospital of Shenzhen Baoan District, No. 118 longjing Second Road, Shenzhen, Guangdong 518101 P.R. China; 2 Department of Medical Records Statistics, University of Chinese Academy of Sciences-Shenzhen Hospital, Shenzhen, Guangdong 518106 P.R. China

**Keywords:** Nasopharyngeal carcinoma, MicroRNA-384, Smad5, Wnt/β-catenin signaling pathway, Proliferation, Apoptosis

## Abstract

Nasopharyngeal carcinoma (NPC) is a major otorhinolaryngological disease with limited effective therapeutic options. This work focused on the function of microRNA-384 (miR-384) on the NPC pathogenesis and the molecules involved. miR-384 expression in cancer tissues and cells was detected. Gain- and loss-of-functions of miR-384 were performed to identify its role in NPC progression. The target mRNA of miR-384 was predicted on an online system and validated through a luciferase reporter assay. The activity of Wnt/β-catenin signaling was detected. Consequently, miR-384 was found to be poorly expressed in NPC tissues and cell lines and was linked to unfavorable survival rates in patients. Overexpression of miR-384 in 6-10B cells suppressed growth, migration, invasion and resistance to apoptosis of cells, but inverse trends were presented in C6661 cells where miR-384 was downregulated. miR-384 targeted Smad5 mRNA. Upregulation of Smad5 counteracted the roles of miR-384 mimic in cells. The NPC-inhibiting effects of miR-384 mimic were also blocked by Wnt/β-catenin activation. To conclude, miR-384 targets Smad5 and inactivates the Wnt/β-catenin pathway, which exerts a suppressing role in NPC cell behaviors as well as tumor growth in vivo. The findings may offer novel thoughts into NPC therapy.

**Supplementary Information:**

The online version contains supplementary material available at 10.1007/s10616-021-00458-3.

## Background

Initiating from the nasopharyngeal mucosal lining, nasopharyngeal carcinoma (NPC) is an epithelial carcinoma which is especially prevalent in east and southeast Asia (Chen et al. 2019). Approximately 129,079 newly diagnoses and 72,987 deaths are reported in this geographical scope in 2018 (Evangelista et al. 2019). Intensity-modulated radiotherapy and stereotactic radiotherapy are currently the primary intervention for NPC; however, frequent NPC recurrence occurs owing to the radio-resistance, and the radiotherapy itself brings varying degrees damages to the adjacent normal tissues (Peng et al. 2019). Additionally, chemotherapy is recommended as an adjuvant for NPC treatment in patients at advanced NPC stages (Liu et al. 2018). Most NPC patients are initially diagnosed at late stages that are often poorly differentiated and prone to develop metastasis, which represents the major cause for NPC treatment failure (Xiao et al. 2019). Those patients who did not respond well to chemo- or radio-therapy eventually acquire distant metastasis with unfavorable prognosis (Fan et al. 2020). Therefore, identifying novel therapies intervening cell growth and metastasis is of great potential to improve outcome of NPC patients.

Gene therapy has emerged as novel options for human disease control including cancer treatment. MicroRNAs are the most studied non-coding RNAs with 22 nucleotides in length which post-transcriptionally regulate genes that hold accountable for fundamental cellular processes including cell differentiation and development (Harrandah et al. 2018). Not surprisingly, aberrant expression of miRNAs is closely associated with oncogenic or cancer suppressing properties through the varying downstream protein-coding genes (Adams et al. 2014), which is also true for NPC (Lee et al. 2016). miR-384 has been noted as a cancer suppressor in several human malignancies such as osteosarcoma (Tan et al. 2020) and gastric cancer (Wang 2019). But the exact expression profile and functions of miR-384 in NPC and the involving mechanisms remain unknown. Our study identified Smad5 as a target mRNA of miR-384. Smad5 is a member of Smad family that are implicated in transforming growth factor-β (TGF-β) signaling in tumor development and metastasis (Matsuzaki 2013; Xiao et al. 2010). Blockage in Smad2 has been documented to reduce the epithelial–mesenchymal transition (EMT) of NPC cells (Hu et al. 2015). Smad5 was found to be abundantly expressed in prostate cancer and linked to biochemical recurrence (Li et al. 2019a). Here, we hypothesized that miR-384 could suppress NPC progression through Smad5 inhibition, and gain- and loss-of-functions of miR-384 and Smad5 were performed in both cell and animal models to validate this hypothesis.

## Materials and methods

### Antibodies, regents and vectors

Antibodies used in western blot analysis were against E-cadherin (#16-3249-82, Invitrogen Inc., Carlsbad, CA, USA), Vimentin (#14-9897-80, Invitrogen), Wnt1 (ab15251, Abcam Inc., Cambridge, MA, USA), β-catenin (#8480, Cell Signaling Technologies (CST), Beverly, MA, USA), glyceraldehyde-3-phosphate dehydrogenase (GAPDH, #5174, CST), goat anti-rabbit immunoglobulin G (IgG, 1:5000, ProteinTech Group, Inc., Hubei, China), and antibodies used in immunohistochemistry (IHC) were against Ki67 (#14-5698-82, Invitrogen) and goat anti-rabbit IgG. A Wnt//β-catenin-specific antagonist IWR-1 (HY-12,238, MedChemExpress, NJ, USA) was dissolved in dimethyl sulphoxide (DMSO) solution to a centration of 1 mM and preserved at −20 °C in the dark. The miR-384 mimic/mimic control, miR-384 inhibitor/inhibitor control, lentiviral vector (LV) and LV-overexpressing Smad5 were purchased from Sangon Biotech Co. Ltd. (Shanghai, China).

### Clinical sample collection

NPC and paracancerous tissues were collected from 43 NPC patients (32 males and 11 females) who were diagnosed and treated in People’s Hospital of Shenzhen Baoan District from August 2013 to May 2014 at a medium age of 47.23 (ranging from 27 to 79) years. A 5-year follow-up study was performed to record the prognosis of patients at a 3-month interval. The inclusion criteria were (1) patients were pathologically diagnosed as NPC; (2) all the malignancies were primary; (3) patients had never underwent radiotherapy or chemotherapy before surgery; (4) patients had complete clinical information. Patients who had (1) chronic system diseases; (2) other malignant tumors or (3) metabolic diseases were excluded. Signed informed consent was acquired from each eligible participant. The study was ratified by the Clinical Ethical Committee of People’s Hospital of Shenzhen Baoan District and in line with the *Helsinki Criteria*.

### Cell transfection

Immortalized nasopharyngeal epithelial cell line NP69 and 4 NPC cell lines C6661, SUNE1, SUN2 and 6-10B and 293T cells were acquired from American Type Culture Collection (Manassas, USA). NPC cells and NP69 cells were cultured in 10 % fetal bovine serum (FBS)-supplemented Roswell Park Memorial Institute-1640 (Gibco Company, Grand Island, NY, USA), while 293T cells were cultured in Dulbecco’s modified Eagle’s medium (DMEM, Gibco) supplemented with 10 % FBS and 1 % penicillin-streptomycin. All cells were cultured at 37 °C with 5 % CO_2_.

Well-growing 6-10B cells and C6661 cells were used for the subsequent experiments. The 6-10B cells were allocated into (1) Mock group (cells were transfected with 100 ng mimic control); (2) miR-384 group (cells were transfected with 100 ng miR-384 mimic); (3) miR-384 + LV-NC (cells were transfected miR-384 mimic and empty LV); (4) miR-384 + Smad5 (LV-NC refers to lentiviral vector-negative control cells were transfected with miR-384 mimic and LV overexpressing Smad5).

The C6661 cells were allocated into InC group (transfected with 100 ng inhibitor control), inhibitor group (transfected with 100 ng miR-384 inhibitor), inhibitor + DMSO group (cells transfected with miR-384 inhibitor were further transfected with DMSO), and inhibitor + IWR-1 group (cells transfected with miR-384 inhibitor were further given 10 nM Wnt/β-catenin antagonist IWR-1). The transfection efficiency was determined 48 hours after transfection using reverse transcription quantitative polymerase chain reaction (RT-qPCR). Well-transfected cells were harvested for the subsequent experiments.

### 5-ethynyl-2’-deoxyuridine (EdU) labeling assay

An EdU labeling assay was conducted to evaluate cell proliferation. The cells were incubated in 6-well plates with EdU solution (10 µM, C0071L, Beyotime Biotechnology Co., Ltd., Shanghai, China), washed in phosphate-buffered saline (PBS), and fixed in 4 % paraformaldehyde for 20 minutes. Then, the cells were washed in 3 % bovine serum albumin (BSA)-supplemented PBS, and then incubated in 0.3 % Triton X-100 for 10 minutes. Next, the plates were filled with Click Additive Solution and incubated in dark environment for 30 minutes, and then filled with Hoechst 3334 (C1022, Beyotime) for 10 minutes of cell incubation. Then, the cells were photographed under a fluorescence microscope (Olympus Optical Co., Ltd. Tokyo, Japan) with 3 fields randomly selected. The proliferation rate (EdU-positive rate) of cells was evaluated as follows: proliferation rate = proliferating cells (EdU-labeled cells)/total cells (Hoechst 33,342-stained cells) × 100 %.

### Acridine orange/ethidium bromide (AO/EB) staining

Exponentially growing C6661 and 6-10B cells were sorted on sterile cover slides for 24 hours and incubated with the mixture of 1 mL 100 mg/mL AO and 100 mg/mL EB (#E607308, Sangon). Then, the cell shape changes were observed under the fluorescence microscope at a × 100 magnification. The proportion of dead cells was evaluated as follows: death rate (%) = dead cells/total cells.

### Cell apoptosis detection

Cell apoptosis was first analyzed using a fluorescein isothiocyanate (FITC)-Annexin V apoptosis kit (BD Biosciences, San Jose, CA, USA). In brief, the C6661 and 6-10B cells were resuspended in 1 × Annexin V binding buffer supplemented with 10 mM HEPES/NaOH (pH = 7.4) (1 × 10^6^ cells/mL). The cells were cultured with FITC-Annexin V (5 µL) and propidium iodide (5 µL) at 37 °C for 20 minutes and analyzed using a flow cytometer (BD Biosciences).

Apoptosis of cells was further examined using Hoechst 33,258 staining. In short, the cells were seeded in 6-well plates and cultured in medium at 37 °C for 24 hours. Then, the cells were trypsinized and centrifuged at 1000 rpm for 5 minutes. After PBS washes, the cells were stained with 300 µL Hoechst 33,258 (C1017, Beyotime) at room temperature for 5 minutes and then observed under the fluorescence microscope at a wavelength of 365 nm. Three random fields were observed at a × 20 magnification for cell apoptosis detection.

### RT-qPCR

Total RNA of cells was extracted using the TRIzol Reagent (Invitrogen). RT was performed using the miRNA and mRNA reverse transcriptase (Promega, Madison, WI, USA) and the random primer (Promega), or using the Bulge-Loop miR-specific RT (RiboBio Co., Ltd., Guangzhou, Guangdong, China). Then, real-time PCR was performed using SYBR Green qPCR Super Mix-UDG (Invitrogen) on a CFX96 Touch System (Bio-Rad, Hercules, CA, USA). GAPDH was set as an internal control for mRNA and U6 for miRNA. Relative RNA expression was evaluated using the 2^−ΔΔCT^ method. The sequences of miR-384 (#HP300350), Smad5 (#HP226654) and U6 (#HP214887) were acquired from OriGene Biotech Co. Ltd. (Beijing, China) and the sequence of GAPDH (#B662104) were from Sangon Biotech Co. Ltd. (Shanghai, China).

### Transwell assay

Transwell assays were conducted to evaluate migration and invasion of cells. For migration detection, each basolateral chamber was loaded with 600 µL 10 % FBS-DMEM, while each apical chamber was loaded with serum-free medium containing 2 × 10^4^ cells. The invasion detection was performed in a similar manner with an additional precoating of Matrigel (BD Biosciences) on the apical chambers. The chambers well placed in a 37 °C constant incubator with 5 % CO_2_. During the incubation, cells on the upper membrane in the apical chambers migrated or invaded (in the setting of Matrigel pre-coating) into the lower surface of the membrane. After 24 h of incubation, the cells invaded or migrated into the lower membrane were stained, fixed in 4 % paraformaldehyde and stained with 0.1 % crystal violet, and counted under a microscope (CX41, Olympus) with at least four random fields selected. The average value of migrated or invaded cells was calculated.

### Dual luciferase reporter gene assay

The binding site of miR-384 and Smad5 mRNA was predicted on StarBase (http://starbase.sysu.edu.cn/). Then the wide type (WT) sequence and the corresponding mutant type (MUT) sequence based on the predicted binding site between miR-384 and 3’UTR of Smad5 were inserted into the pMIR-RB-REPORT™ vectors (RiboBio) to construct pMIR-RB-REPORT^TM^-Smad5-WT and Smad5-MUT vectors. Well-designed WT and MUT vectors were co-transfected with mimic control or miR-384 mimic into HEK-293T cells. Cells were lysed 48 hours later, and the relative luciferase activity was detected using a luciferase assay kit (RG005, Beyotime Biotechnology Co., Ltd. Shanghai, China).

### Western blot analysis

Total protein in cells was extracted using radio-immunoprecipitation assay cell lysis buffer (Beyotime) containing ethylene diamine tetraacetic acid-proteinase inhibitor (Roche Ltd., Basel, Switzerland). The protein concentration was determined using a bicinchoninic acid kit (Thermo Scientific Pierce, Rockford, IL, USA). The protein extracts were run on 4–20 % SDS-PAGE and transferred onto polyvinylidene fluoride membranes (Merck Millipore, Billerica, MA, USA). Next, the membranes were blocked in 5 % non-fat milk and co-cultured with the primary antibodies at 4 °C overnight, and then with the secondary antibody at room temperature for 1 hour. The protein bands were visualized using an enhanced Chemiluminescence System (Thermo Fisher).

### Xenograft tumors in nude mice

Ten BALB/c nude mice (6 weeks old, 20 ± 2 g, SLAC Laboratory Animal Co., Ltd., Shanghai, China) were randomly assigned into two groups, 5 in each. Then, each mouse was subcutaneously injected with 0.1 mL FBS-free RPMI-1640 containing 1 × 10^5^ cells with stable transfection of miR-384 mimic or mimic control (Mock). The mice were euthanized by an overdose of pentobarbital sodium (120 mg/kg) 5 weeks after injection. The xenograft tumors were taken out and the width (a) and length (b) of tumor were measured, and the volume (V) was calculated as the following formula: V = (a^2^ × b)/2. Animal studies were performed in accordance with the principles and procedures approved by the Committee on the Ethics of Animal Experiments of People’s Hospital of Shenzhen Baoan District. Great attempts were made to minimize the usage and pain of animals.

### IHC staining

Tumor tissues from mice were embedded in paraffin, dewaxed, and hydrated in ethanol to distilled water. The sections were incubated with hydrogen peroxide and blocked with goat serum (Beyotime), and then incubated with 50 µL anti-Ki67 at 4 °C overnight. Next, the sections were washed with PBS, and then incubated with biotinylated secondary antibody anti-IgG at 37 °C for 30 minutes. After that, the samples were co-incubated with streptavidin-biotin-peroxidase complex (1:200, Sigma-Aldrich Chemical Company, St Louis, MO, USA). The staining was developed by 3,3’-diaminobenzidine, and the nuclei were counter-stained with hematoxylin for 30 seconds.

### Statistical analysis

Data were analyzed using SPSS 22.0 (IBM Corp. Armonk, NY, USA). Data are presented as mean ± standard deviation (mean ± SD) from three individual experiments. Differences between two groups were measured using the *t*-test, while differences among multiple groups were analyzed using one-way or two-way analysis of variance (ANOVA). Tukey’s multiple comparisons test was used for the pairwise comparison after ANOVA analysis. Survival curve was drawn via the Kalpan-Meier method and analyzed using log rank test. *p* was obtained by two-tailed test and *p* < 0.05 was regarded to show a statistically significant difference.

## Results

### Poor expression of miR-384 indicates unfavorable prognosis in NPC patients

**Fig. 1:**
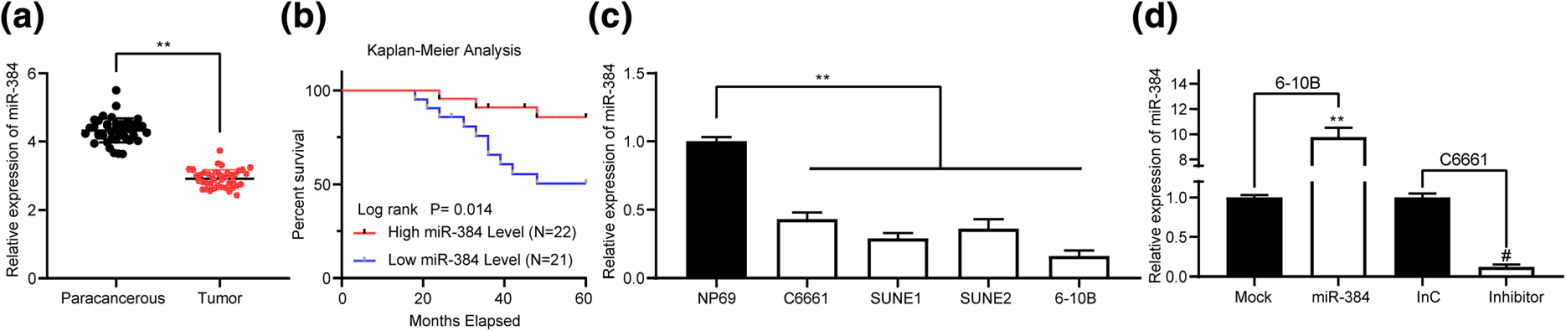
Poor expression of miR-384 indicates unfavorable prognosis in NPC patients.** a** miR-384 expression in 43 pairs of NPC and paracancerous tissues determined by RT-qPCR;** b** the correlation between miR-384 expression and survival rate of NPC patients evaluated by Kapla-Meier analysis;** c** miR-384 expression in NP69 and in NPC cell lines (C6661, SUNE1, SUNE2 and 6-10B) determined by RT-qPCR; **d**, 6-10B with lowest miR-384 expression among the four NPC cell lines was transfected with miR-384 mimic or mimic control, while C6661 cells with highest miR-384 expression was given miR-384 inhibitor or inhibitor control, after which miR-384 expression in cells was measured by RT-qPCR. Data are exhibited as mean ± SD from three independent experiments; Data in panel **a** were analyzed using paired *t* test, while data in panels** c**, **d** were analyzed using one-way ANOVA and Tukey’s multiple comparison test; **p < 0.01 vs. paracancerous tissues or the NP69 group; #*p* < 0.05 vs. the InC group

At first, RT-qPCR was performed to determine miR-384 expression in 43 pairs of NPC tissues and paracancerous tissues, which identified that miR-384 expression was lower in cancer tissues relative to the paracancerous ones (Fig. 1a). The medium value of miR-384 expression was 2.96, based on which the patients were allocated into high miR-384 level group (> 2.96) and low miR-384 level group (< 2.96), respectively. It was noteworthy that patients with a higher miR-384 level had a higher 5-year survival rate (Fig. 1b). In addition, the clinical baseline characteristics of patients were collected and analyzed, which suggested that high expression of miR-384 was correlated with reduced lymph node metastasis and tumor node metastasis (TNM) staging while increased tumor differentiation (Supplementary Table 1). Next, miR-384 expression in NP69 and in NPC cell lines was detected. It was found that miR-384 expression was lower in NPC cell lines (C6661, SUNE1, SUNE2 and 6-10B) than that in NP69 cells (Fig. 1c). Among the NPC cell lines, the C6661 cells with the highest miR-384 expression, and the 6-10B cells with the lowest miR-384 expression, were collected for the subsequent experiments. Then, miR-384 mimic or the mimic control was transfected into 6-10B cells, while miR-384 inhibitor or the inhibitor control was transfected into C6661 cells, and RT-qPCR results confirmed that 6-10B cell line overexpressing miR-384 and C6661 cell line silencing miR-384 were successfully established (Fig. 1d).

### Overexpression of miR-384 inhibits viability of NPC cells

**Fig. 2:**
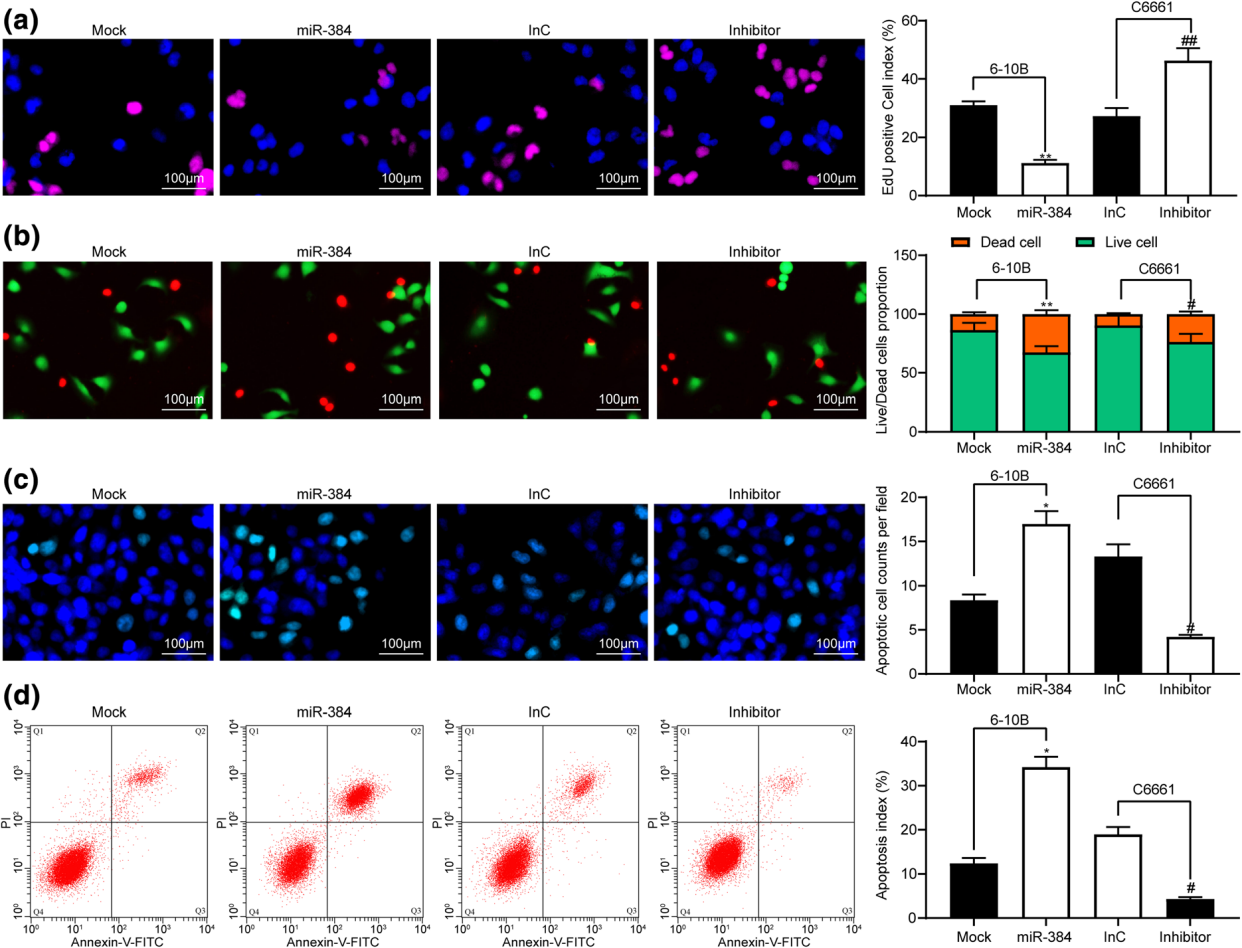
Overexpression of miR-384 inhibits viability of NPC cells. **a** proliferation activity of 6-10B and C6661 cells determined by EdU labeling assay; **b** proportion of live and dead cells measured by AO/EB staining; **c** number of apoptotic cells determined by Hoechst 33,258 staining; **d** ratio of apoptotic cells detected by flow cytometry. Data are exhibited as mean ± SD from three independent experiments; Data were analyzed using one-way ANOVA and Tukey’s multiple comparison test; *p < 0.05; **p < 0.01 vs. the Mock group; #*p* < 0.05, ## *p* < 0.01 vs. the InC group

Following the findings above, we further explored the direct roles of miR-384 in NPC cell behaviors. The EdU labeling assay showed that overexpression of miR-384 led to a notable suppression in the proliferation activity in 6-10B cells, while the proliferation of C6661 cells was increased upon miR-384 silencing (Fig. 2a). In addition, the proportion of live/dead cells detected by AO/EB staining suggested that miR-384 mimic transfection reduced the proportion of live cells in 6-10B cells, while miR-384 inhibitor resulted in inverse trends (Fig. 2b). In concert with this, the Hoechst 33,258 staining and flow cytometry results also showed that overexpression of miR-384 reduced the number of apoptotic cells while downregulation of miR-384 led to a decline in cell apoptosis (Fig. 2c, d).

### miR-384 mimic inhibits EMT, migration and invasion of NPC cells

**Fig. 3:**
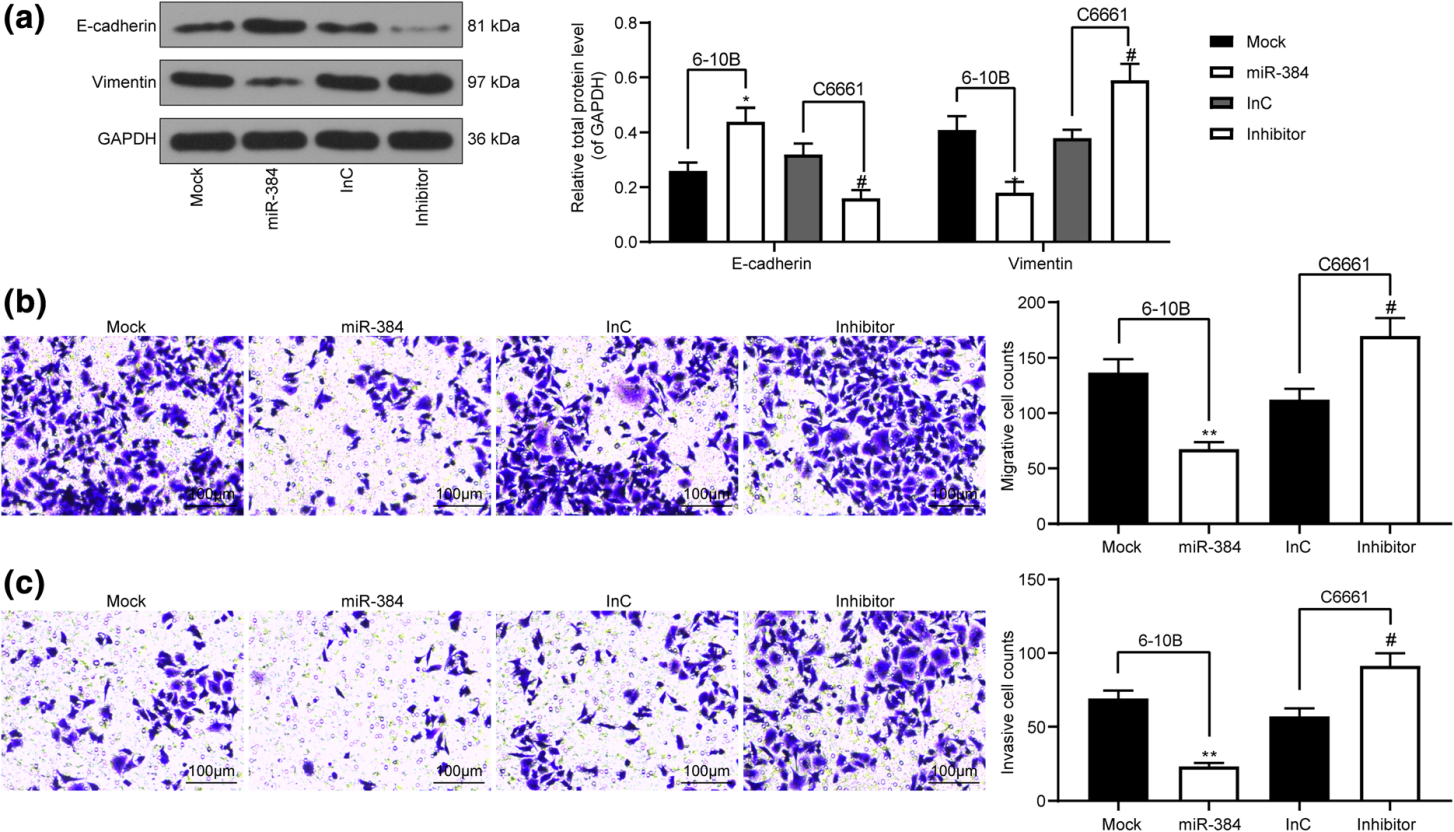
miR-384 mimic inhibits EMT, migration and invasion of NPC cells. **a** levels of mesenchyme marker protein Vimentin and epithelium-marker protein E-cadherin in cells measured by western blot analysis; **b**, **c** number of migrated (**b**) and invaded (**c**) cells determined by Transwell assays. Data are exhibited as mean ± SD from three independent experiments; in panel (**a**), data were analyzed using two-way ANOVA, while data in panels (**b**) and (**c**) were analyzed by one-way ANOVA, and Tukey’s multiple comparison test was used for the post-hoc test after ANOVA; *p < 0.05; **p < 0.01 vs. the Mock group; #*p* < 0.05 vs. the InC group

We further evaluated the levels of mesenchyme marker protein Vimentin and epithelium-marker protein E-cadherin in cells using western blot analysis. The results showed that overexpression of miR-384 increased the level of E-cadherin while silencing of miR-384 increased the level of Vimentin in cells (Fig. 3a). In addition, the Transwell assays found that miR-384 mimic administration reduced the number of migrated and invaded cells, and accordingly, these trends were reversed in C6661 cells where miR-384 was suppressed (Fig. 3b, c).

### miR-384 targets Smad5 to induce the Wnt/β-catenin signaling defect

**Fig. 4:**
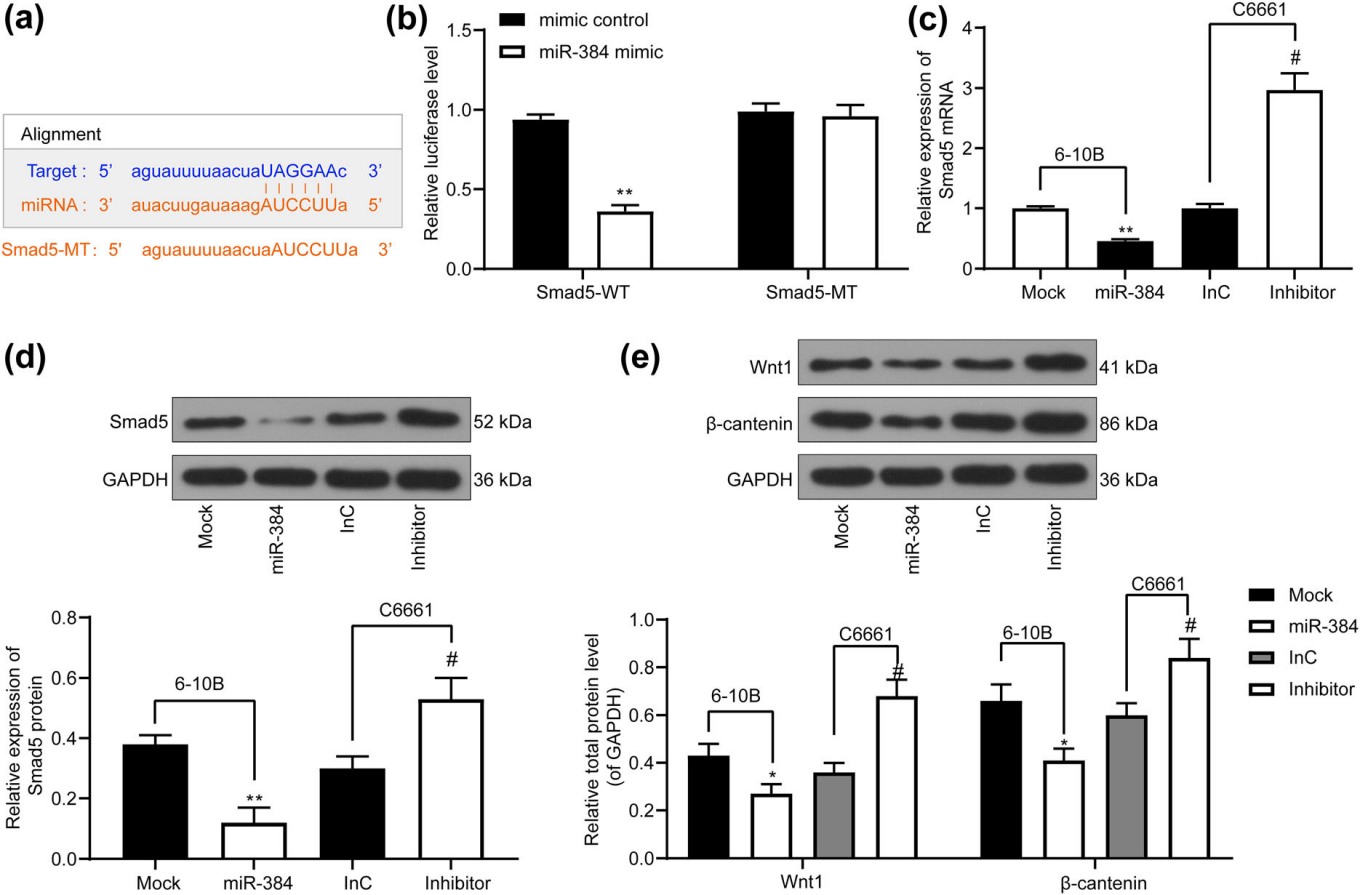
miR-384 inhibits Smad5 expression to inactivate the Wnt/β-catenin signaling pathway. **a**, **b**, binding relationship between miR-384 and Smad5 predicted on StarBase (http://starbase.sysu.edu.cn/) and validated through a dual luciferase reporter gene assay; **c**, **d**, mRNA (**c**) and protein (**d**) expression of Smad5 assessed by RT-qPCR and western blot analysis, respectively; (**e**), expression of Wnt1 and β-catenin in cells determined by western blot analysis. Data are exhibited as mean ± SD from three independent experiments; in panels (**b**) and (**e**), data were analyzed using two-way ANOVA, while data in panels (**c**) and (**d**) were analyzed by one-way ANOVA, and Tukey’s multiple comparison test was used for the post-hoc test after ANOVA; *p < 0.05; **p < 0.01 vs. the Mock group; #*p* < 0.05 vs. the InC group

The findings above triggered us to figure out the downstream mechanisms. The StarBase predicted that miR-384 directly binds to the 3’UTR of Smad5 mRNA sequence (Fig. 4a). This putative binding relationship was verified by a luciferase assay. It was found that the activity of Smad5-WT luciferase reporter vector was significantly reduced in 293T cells when co-transfected with miR-384 mimic, while the luciferase activity in cells transfected with Smad5-MUT vector or mimic control showed little change (Fig. 4b). Furthermore, RT-qPCR and western blot analysis identified a negative association between miR-384 expression and Smad5 expression in 6-10B and C6661 cells (Fig. 4c, d). In addition, the Wnt/β-catenin is closely associated with cancer progression including NPC (Pang et al. 2019). Then, our study found that miR-384 mimic inhibited the expression of Wnt1 and β-catenin in cells, and miR-384 inhibitor, correspondingly, led to reversed trends (Fig. 4e).

### Overexpression of Smad5 partially blocks the effects of miR-384 mimic on NPC cells

**Fig. 5:**
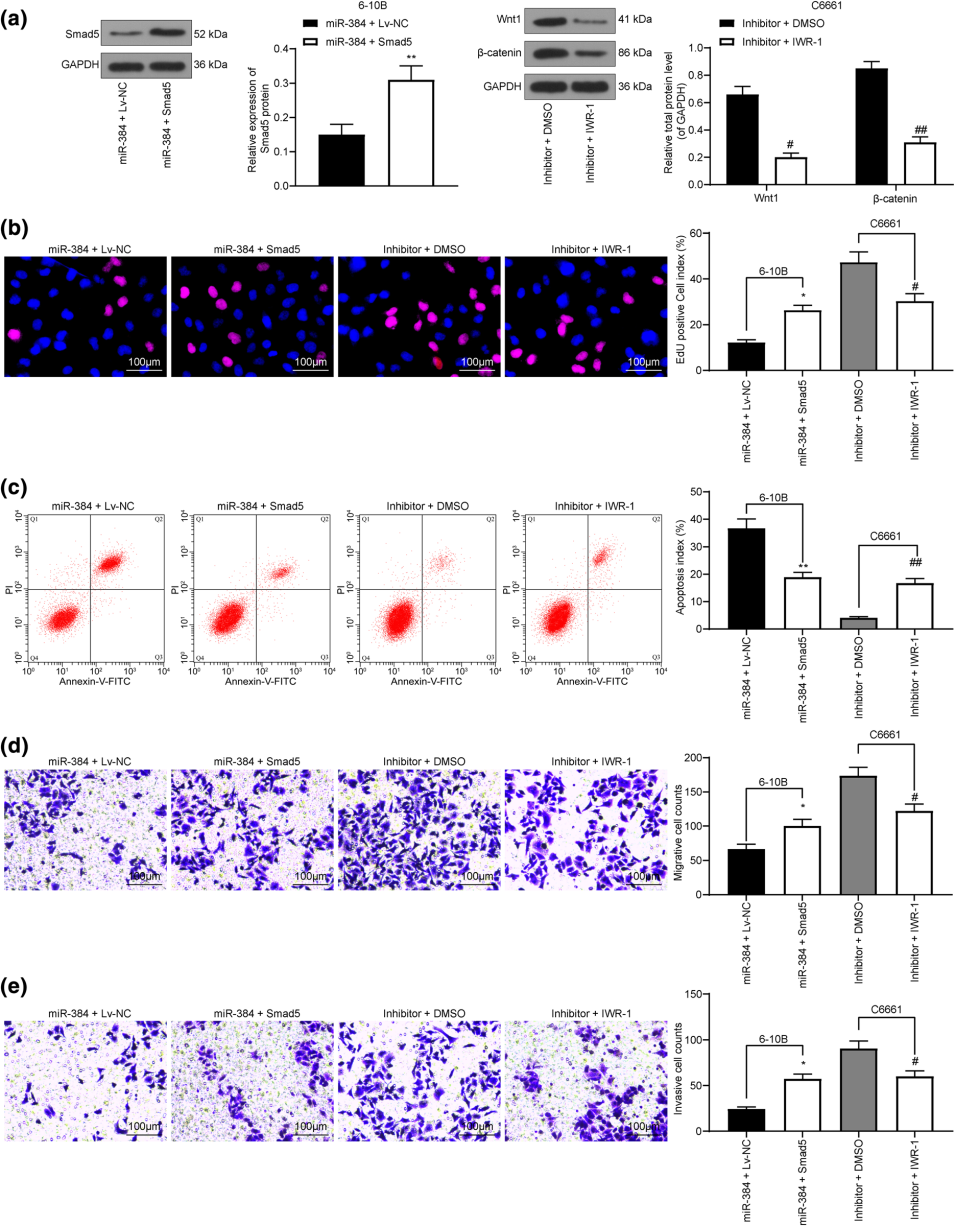
Overexpression of Smad5 or activation of β-catenin partially blocks the effects of miR-384 on NPC cells. LV overexpressing Smad5 was transfected into 6-10B cells pre-transfected with miR-384 mimic, while a Wnt/β-catenin-specific antagonist, IWR-1, was introduced into C6661 cells with silenced miR-384. **a** Smad5 expression in Smad5 cells and Wnt/β-catenin activation in C6661 cells determined by western blot analysis; **b** proliferation ability of cells determined by EdU labeling assay; **c** ratio of apoptotic cells determined by flow cytometer; **d**, **e**, number of migrated (**d**) and invaded (**e**) cells measured by Transwell assays. Data are exhibited as mean ± SD from three independent experiments; in panel (**a**), data were analyzed using unpaired t test or two-way ANOVA, while data in panels **b**, **c**, **d** and **e** were analyzed by one-way ANOVA, and Tukey’s multiple comparison test was used for the post-hoc test after ANOVA; *p < 0.05; **p < 0.01 vs. the miR-384 + Lv-NC group; #*p* < 0.05, ##*p* < 0.01 vs. the Inhibitor + DMSO group

To further confirm the involvement of inhibition of Smad5 and β-catenin in the miR-384-mediated events, 6-10B cells with high expression of miR-384 were further transfected with LV overexpressing Smad5, while a Wnt/β-catenin-specific antagonist, IWR-1, was introduced into C6661 cells where miR-384 was suppressed. The successful transfection and drug administration were identified by western blot analysis (Fig. 5a). Then, the EdU labeling assay results showed that the proliferation of 6-10B cells suppressed by miR-384 mimic was recovered after Smad5 overexpression, while artificial inactivation of the Wnt/β-catenin signaling led to a decline in proliferation activity in C6661 cells (Fig. 5b). Likewise, apoptosis rate in 6-10B cells, which was initially enhanced by miR-384 mimic, was inhibited following further Smad5 overexpression. Accordingly, the apoptosis of C6661 cells was notably increased by IWR-1 (Fig. 5c). In addition, Smad5 enhanced the invasion and migration abilities of 6-10B cells, while the migration and invasion potentials of C6661 cells was inhibited upon Wnt/β-catenin inactivation (Fig. 5d, e).

### Overexpression of miR-384 inhibits growth of xenograft tumor in vivo

**Fig. 6:**
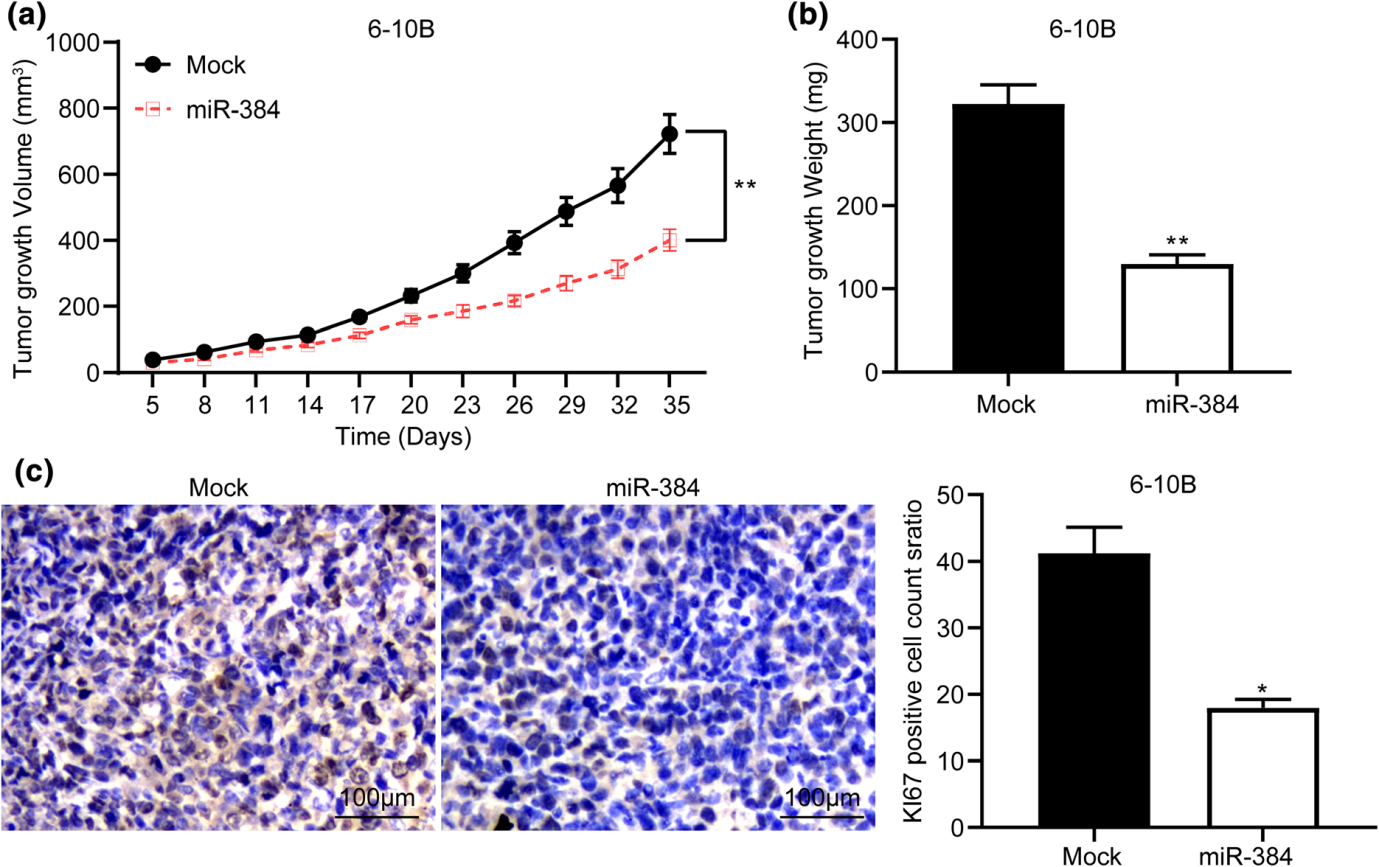
Overexpression of miR-384 inhibits growth of xenograft tumor in vivo. 6-10B cells with stable miR-384 mimic or mimic control transfection were implanted in nude mice. **a** change of tumor volume after cell implantation in mice; **b** tumor weight in mice on the 35th day after animal euthanasia; **c** Ki-67 expression in mouse tumors detected by IHC staining; Repetition = 3. Data are exhibited as mean ± SD; n = 5 in each group; in panel **a**, data were analyzed using two-way ANOVA and Tukey’s multiple comparison test, while data in panels **b** and **c** were analyzed by the unpaired *t* test; *p < 0.05; **p < 0.01 vs. Mock group

Xenograft tumors were induced in nude mice to further study the role of miR-384 in NPC growth in vivo. In brief, 6-10B cells with stable transfection of miR-384 mimic or mimic control were implanted in nude mice through subcutaneous injection. It was found that overexpression of miR-384 reduced the growth rate in mice (Fig. 6a) and reduced the tumor weight on the 35th day (Fig. 6b). Thereafter, the tumor tissues were cut into sections for IHC staining, and the results suggested that overexpression of miR-384 reduced the rate of positive Ki67 expression in tumors (Fig. 6c).

## Discussion

NPC treatment is still a major challenge among all otorhinolaryngological diseases with limited effective therapeutic options. Owing to the complex anatomic structure in the nasopharyngeal region, surgery is rarely performed in NPC treatment, and chemotherapy is likely to bring severe side-effects, while even the currently most effective radiotherapy only results in a 50–70 % 5-year survival rate of NPC patients (Wang et al. 2019a). In the current study, we identified miR-384 as a potential option for molecule therapy targeting NPC. This miRNA was found to inhibit the malignant behaviors including proliferation, invasion and migration of NPC and the tumor growth in vivo, during which the down-regulation of Smad5 and Wnt/β-catenin signaling was possibly involved.

The initial finding of this paper was that miR-384 was poorly expressed in the NPC tumor tissues relative to the paired paracancerous tissues, and patients with relative higher levels of miR-384 owned a higher 5-year survival rate. These results preliminarily showed a potential beneficiary role of miR-384 in NPC. Then, altered miR-384 was introduced in NPC 6-10B and C6661 cell lines, after which we found 6-10B cells with overexpressed miR-384 presented decreased proliferation activity, migration, invasion, and increased apoptosis, and these trends were reversed in C6661 cells with silenced miR-384. In addition, miR-384 overexpression reduced the EMT activity in cells, another major characteristic during cancer progression, presenting by decreased Vimentin while increased E-cadherin (Paolillo and Schinelli 2019). miRNAs are well-known to function as tumor suppressor genes or oncogenes in NPC by modulating specific target genes which are implicated in multiple cellular processes and pathways (Bruce and Liu 2014; Lee et al. 2016). For instance, knockdowns of miR-534 (Jiang et al. 2019) and miR-194 (Guo et al. 2019a) have been documented to inhibit NPC progression. Some miRNAs such as miR-449b-5p (Yin et al. 2019) and miR-184 (Zhu et al. 2018) have been noted to exert suppressing functions in NPC pathogenesis. As for miR-384, its cancer-inhibiting roles have been validated in many cancer types, such as inducing apoptosis of non-small cell lung cancer cells (Guo et al. 2019b) and inhibiting growth and metastasis of osteosarcoma cells (Wang et al. 2019b). More relevantly, miR-384 has been noted as an RNA sponge for lncRNA TUG1 and blocked the promoting role of lncRNA TUG1 in NPC cell viability, proliferation and EMT (Qian et al. 2019). Here, our paper validated the tumor-suppressing role of miR-384 in NPC cells and in vivo. Moreover, we had the further involved potential molecules figured out.

Following the findings above, we identified Smad5 as a potential target of miR-384 in NPC through integrated online prediction and a dual luciferase reporter assay. Since Smad5 has been suggested as an oncogene in several tumors including Epstein–Barr virus (EBV)-associated gastric cancer (Jing et al. 2018). EBV is linked to multiple human cancers, and in particular, its closest correlation is well-known with undifferentiated NPC (Tsao et al. 2017). Specifically, Smad5 has been documented to be indirectly up-regulated by lncRNA Smad5-AS1, which promoted the EMT and malignancy of NPC cells (Li et al. 2019b; Zheng et al. 2019). The miR-384-Smad5 axis, to the best of knowledge, has never been investigated. Here, we first validated that Smad5 was highly expressed when miR-384 was inhibited. Additionally, we evidenced that overexpression of Smad5 partially recovered proliferation, invasion, migration and resistance to apoptosis of 6-10B cells that were inhibited by miR-384, indicating that Smad5 inhibition is possibly involved in miR-384-mediated events.

The Wnt/β-catenin pathway participates in many fundamental cellular functions like organ formation, stem cell renewal and cell survival, and specifically, is activated in multiple human cancers including NPC (Krishnamurthy and Kurzrock 2018; Liu et al. 2015). Here, our study noted that the Wnt/β-catenin signaling defect was also induced following miR-384 overexpression. Since we found miR-384 mimic led to a significant decline in Wnt1 and β-catenin expression. Accordingly, the strengthened malignancy in C6661 cells which pre-transfected with miR-384 inhibitor was partially blocked following the artificial Wnt/β-catenin inactivation by IWR-1. This might further explain that miR-384 inhibits NPC cell proliferation, potentially, through inactivating the Wnt/β-catenin pathway.

## Conclusions

To sum up, our present study evidenced that miR-384 might serve as a hopeful biomarker in NPC patients since it was positively correlated with NPC patient prognosis, and it showed significant tumor-inhibiting effects in both cell and animal experiments. During the process of miR-384-mediated events, down-regulation of Smad5 and the Wnt/β-catenin pathway was implicated. However, the current study provided limited information about the link between Smad5 inhibition and the Wnt/β-catenin inactivation. We hope more studies will be conducted in this field to validate our findings and to identify more novel molecules in NPC development.

## Electronic Supplementary Material

Below is the link to the electronic supplementary material.

Electronic supplementary material 1 (DOCX 17 kb)
