# Supplementary material for: MicroRNA-384 inhibits nasopharyngeal carcinoma growth and metastasis via binding to Smad5 and suppressing the Wnt/β-catenin axis
Source: Cytotechnology. 2021 Feb 26;73(2):203–15. doi: 10.1007/s10616-021-00458-3 (PMC8035371; doi:10.1007/s10616-021-00458-3)
Supplement: Supplementary file 1 — Electronic supplementary material 1 (DOCX 17 kb) [file 10616_2021_458_MOESM1_ESM.docx]

**Supplementary Table 1** Clinical baseline characteristics of patients with NPC

| Characteristics | N = 43 | miR-384 expression | | *p* value |
| --- | --- | --- | --- | --- |
|  |  | High (n = 29) | Low (n =31) |  |
| Gender |  |  |  |  |
| male | 28 | 16 | 12 | 0.723 |
| Female | 15 | 6 | 9 |  |
| Age |  |  |  |  |
| < 50 | 19 | 9 | 10 | 0.346 |
| > 50 | 24 | 13 | 11 |  |
| TNM stage |  |  |  |  |
| I-II | 12 | 10 | 2 | 0.016 |
| III-IV | 31 | 12 | 19 |  |
| Lymph node metastasis |  |  |  |  |
| N0 | 11 | 9 | 2 | 0.034 |
| N1-3 | 32 | 13 | 19 |  |
| Differentiated level |  |  |  |  |
| Well | 20 | 15 | 5 | 0.005 |
| Poor | 23 | 6 | 16 |  |
| Tumor size |  |  |  |  |
| < 5 cm | 16 | 7 | 9 | 0.534 |
| > 5 cm | 27 | 15 | 12 |  |

Note: NPC, nasopharyngeal carcinoma; TNM, tumor node metastasis
